# Blood cytokine pattern and clinical outcome in knee arthroplasty patients: comparative analysis 5 years after standard versus “hypoallergenic” surface coated prosthesis implantation

**DOI:** 10.1080/17453674.2018.1518802

**Published:** 2018-10-29

**Authors:** Peter Thomas, Philipp Hisgen, Hartmuth Kiefer, Ulf Schmerwitz, Andreas Ottersbach, Dominique Albrecht, Burkhard Summer, Christian Schinkel

**Affiliations:** 1 Department of Dermatology and Allergology, Ludwig-Maximilians-University, Munich, Germany;;; 2 Department of Trauma Surgery and Orthopaedics, Klinikum Memmingen, Germany (Academic Teaching Hospital of Ludwig-Maximilians-University Munich);;; 3 Clinic of Trauma Surgery and Orthopaedics, Bünde, Germany;;; 4 Clinic for Orthopaedic Surgery, Brig, Switzerland;;; 5 Clinic of Trauma Surgery and Orthopaedics, St Gallen, Switzerland

## Abstract

Background and purpose — Metal sensitivity might provoke complications after arthroplasty. Correspondingly, coated “hypoallergenic” implants are of interest but long-term follow-up data are missing. Thus, we assessed immunological and clinical parameters in such patients.

Patients and methods — 5 years’ follow-up data were obtained from 3 centers, which used either a standard total knee replacement (TKR) or the identical implant with multilayer surface zirconium nitride based coating. Of the 196 patients (mean age 68 years (44–84), 110 females) 97 had arthroplasty with a coated surface, and 99 were treated by a standard TKR of the same type. Investigations were Knee Society Score (KSS), Knee injury and Osteoarthritis Outcome Score (KOOS), radiographic analysis, and cytokine measurement in peripheral blood. Pro- and anti-inflammatory cytokines were evaluated by cytometric beads assay and RT-PCR.

Results — Survival rate (Kaplan–Meier) was 98% for coated and 97% for uncoated implants after 5 years. Mechanical axis and KSS pain score (42 vs. 41 (0–50)) were comparable. Most serum cytokine levels were comparable, but mean interleukin-8 and interleukin-10 levels were higher in the group with an uncoated implant. IL-8: 37 (SD 7.5) pg/mL vs. 1.1 (SD 4.3) (p < 0.001); IL-10: 3.6 (SD 2.5) vs. 0.3 (SD 1.8) pg/mL (p < 0.001).

Interpretation — There was similar clinical outcome 5 years after standard and surface-coated TKR. In peripheral blood there was an increased pro-inflammatory status, i.e., significant elevation of IL-8 and the anti-inflammatory IL-10, after standard uncoated prosthesis. Any long-term effects of these cytokine changes are unknown.

Revision rates after total knee replacement (TKR) are about 9.5% in Germany and about 8.4% in the USA for the year 2011 (Wengler et al. 2014). The pathogenesis of implant failure is multifactorial (Sharkey et al. 2002, Bourne et al. 2010), and adverse reactions to metal ions released from arthroplasty represent one such factor. Both unspecific (“innate”) immune reactions and specific T lymphocytic sensitization with subsequent hypersensitivity mechanisms may contribute to arthroplasty intolerance reactions in patients (Mahendra et al. 2009, Reito et al. 2016, Hallab et al. 2017).

The clinical spectrum encompasses both local and systemic effects, being often unspecific like: pain, swelling, effusion, reduced range of motion, impaired wound healing, eczema. Diagnostic findings include osteolysis, lymphocyte-dominated peri-implant inflammation, and in extreme situations soft tissue necrosis or pseudotumor formation. In 2017 Hallab et al. framed the statement “a consensus of studies agree the dominant form of this response is due to innate reactivity to implant debris danger signaling” (Hallab et al. 2017). Thus, the corresponding release of cytokines and chemokines might open the way to implant failure. Examples are: chemokines like IL-8, CXCL10, or monocyte chemotactic protein-1 (Lawrence et al. 2014), danger signaling associated inflammasome pathway with IL-1I/IL-18 formation (Yang et al. 2015) or TIRAP/Mal mediated particle-induced osteolysis (Bechtel et al. 2016).

Correspondingly, various approaches have been discussed to diminish the metal ion exposure in arthroplasty patients, such as reducing wear by modifying modularity or surface modification (Bolognesi et al. 2015, Morlock 2015, Thomas et al. 2015). It might be speculated that the mostly inflammatory pathologic reactions to metal ions and debris represent stages of “broken tolerance” of the implant-exposed organism. However, the underlying mechanisms leading to immune homeostasis are also not well understood in patients with symptom-free arthroplasty.

We compared 2 groups of TKR patients at 5-year follow-up who had had the identical type of prosthesis with one exception: In half of the patients a variant was implanted with multilayer Advanced Surface (AS) coating (zirconium nitride (ZrN)) to reduce metal ion release. First, we assessed the clinical performance of the 2 variants of TKR in these patients. Second, we investigated immune reactivity to TKR with a focus on the systemic response, i.e., the response pattern of selected cytokines and chemokines in peripheral blood. The latter was done because, in contrast to in vitro studies often using cell lines, such an approach is rarely performed. We hypothesized that a local yet “subclinical” inflammatory state might be also recognizable by this approach.

## Patients and methods

### Patients and controls

All 322 patients who received a TKR (e.motion UC, Aesculap AG, Tuttlingen, Germany) in 2007 were invited for mid-term follow-up (around 5 years). Of these, 41 patients were lost to follow-up. For 78 patients, who were not willing or able to participate in the follow-up, at least information on prosthesis status could be obtained and in 7 patients 1 or more implant components had been exchanged. 196 patients (mean age 68 years [44–84], 110 females) with primary knee arthroplasty took part in the study (Table). The follow-up examinations were done in 3 centers:

- Klinikum Memmingen (Germany, center 1); here all patients received exclusively the surface-coated variant of the knee implant.
- Lukas Hospital Bᴙnde (Germany, center 2) and Hospital Oberwallis Brig (Switzerland, center 3); here patients routinely received the uncoated variant of the knee implant.

In centers 2 and 3, if patients had a history of metal allergy they received the surface-coated implant. These few patients are not included in this evaluation. All standard knee implants used were CoCr alloys and were cemented. Only patients from center 3 had received retropatellar resurfacing, but no difference in cytokine levels was found between the two centers (2 and 3) with uncoated implants.

The follow-up assessment encompassed clinical, radiological, and immunological examination. Analysis of blood samples was done at the Department of Dermatology and Allergology, Ludwig-Maximilians-University (LMU) Munich. The Table gives the patients’ characteristics.

In addition to the arthroplasty patients, 40 individuals without a metal implant volunteered as blood donors for cytokine control assessment: 20 healthy individuals (median age 61 years [52–63], 14 females; 3 with a history of metal allergy) without a metal implant; 20 individuals with knee osteoarthritis prior to primary arthroplasty (median age 60 years [46–84], 17 females; 14 with a history of metal allergy). The 20 individuals prior to arthroplasty were patients presented by their orthopedic surgeons to the Department of Dermatology and Allergology of LMU for allergy diagnostics. Thus in this group patients with an allergy background are overrepresented. However, we thought that pre-existing “allergy status” would not alter preoperative blood cytokine levels.

### Clinical and radiological assessments

A detailed physical examination was done. In addition, functional and symptom assessment was performed by Knee Society Score (KSS) and Knee injury and Osteoarthritis Outcome Score (KOOS). Radiological evaluation was done using standard weight-bearing knee radiographs in two planes and in load-bearing leg axis to evaluate prosthesis positioning and presence of radiolucencies.

### Analysis of blood cytokine levels and expression

From each patient 2 blood samples were obtained.

*First sample* — After blood drawing, serum was obtained from this sample by centrifugation and immediately frozen. 190 cryopreserved serum samples (98 patients with uncoated, 92 patients with coated arthroplasty) could be analyzed. The blinded samples were assessed for the presence and concentration of 10 cytokines by a multiplex cytometric bead assay (CBA; BD Biosciences, Heidelberg, Germany) via flow cytometry using a FACS canto (BD Biosciences, Heidelberg, Germany). The panel of cytokines included—with the restriction of sometimes overlapping functions—inflammatory (IL-4, IL-5, IL-6, IL-17A, IL-1I, IFN©, TNF↑), chemoattractant (IL-8, CXCL10), and immune-regulating (IL-10) factors. The respective detection limits were <0.5 pg/mL.

*Second sample* — A peripheral blood sample with EDTA was stabilized with RNAlater (Ambion, Carlsbad, USA) and cryopreserved. Then RNA was isolated with the RiboPure Blood Kit (Ambion, Carlsbad, USA). After reverse transcription into cDNA (Transcriptor First strand cDNA Synthesis Kit, Roche, Mannheim, Germany) gene expression of 5 different genes (IFN©, Foxp3, IL-10, IL-8, CASP1) was analyzed by semiquantitative realtime PCR in a LightCycler 1.5.

### Statistics

The statistical packages SPSS (Version 19.0; SPSS Inc, Chicago, IL, USA) and SAS (Version 9.4; SAS Institute, Cary, NC, USA) were used. Differences in the blood cytokine levels were assessed using Student’s t-test. Prosthesis survival was calculated by Kaplan–Meier analysis.

### Ethics, funding, and potential conflicts of interest

The study was approved by the local ethics committee (approval number 186-12). Patient examination and blood sampling was done after obtaining informed consent. Funding was by a research grant from Aesculap AG. There was no further conflict of interest.

## Results

### Orthopedic and radiologic outcomes

The average time of follow-up in the 196 arthroplasty patients was 5.7 years (54–77 months). The mean BMI was 28 (20–44) and was comparable between the groups. The KSS were similar between the centers and groups: Center 1 (patients with surface-coated TKR): mean 84 (SD 15). Center 2 (patients with uncoated TKR): mean 82 (SD 19). Center 3 (patients with uncoated TKR): mean 85 (SD 15).

Radiolucent lines were investigated by plain radiographs. No complete loosening was observed. The revision free implant survival rates after 5 years (Kaplan–Meier) were comparable with 98% for coated and 97% for uncoated implants.

### Cytokine levels and expression in peripheral blood

**Figure 1:**
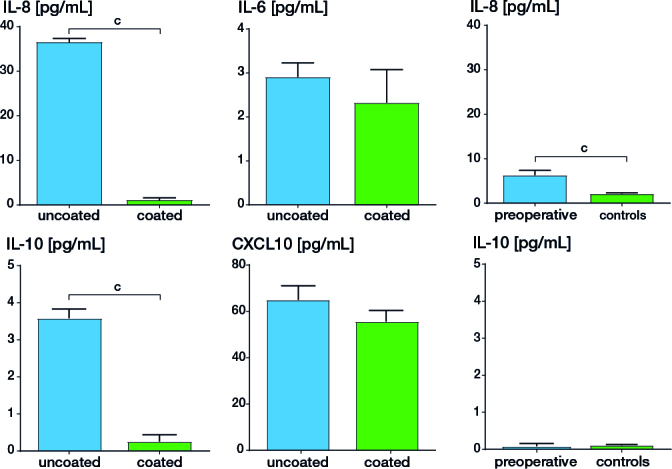
(left panel) Serum cytokine levels in patients with the uncoated (n = 98) and coated (n = 92) TKR (e.motion/e.motion-AS). (right panel) Serum cytokine levels in 20 patients prior to primary arthroplasty (“preoperative”) and 20 healthy individuals without implant (“controls”). The cytokines IL8, IL-6, the immunoregulatory IL-10, and the T-cell chemoattractant CXCL10 were assessed. Concentrations are given in pg/mL. **^b^** p < 0.01; **^c^** p < 0.001; standard deviation is given.

Blood samples of 190 patients could be evaluated. Marked differences were found for 3 of the 10 cytokines. There was a clear-cut difference between patients with uncoated and coated arthroplasty in circulating level of: (i) IL-8 (37 pg/mL [SD 5.6] vs. 1.2 pg/mL [SD 2.1]; p < 0.001); (ii) IL-10 (3.6 pg/mL [SD 8.9] vs. 0.25 pg/mL [SD 0.5]; p < 0.001); (iii) and to a lesser extent IL-6 (2.9 pg/mL [SD 2.0] vs. 2.3 pg/mL [SD 3.4]; p < 0.05). Levels of CXCL10 did not differ statistically significantly (64.9 pg/mL, SD 39.0 vs. 55.6 pg/mL, SD 29.9). The osteoarthritis control patients (prior to arthroplasty) also showed a less pronounced IL-8 and CXCL-10 elevation as compared with the healthy controls. With regard to IL-8 and IL-10, the serum cytokine levels of the healthy controls were similar to those of the patients with a coated arthroplasty. When comparing results of patients with allergy background (here mostly “atopy”) with those without such background, no major difference was found in the different groups. The data are summarized in Figure 1.

**Figure 2:**
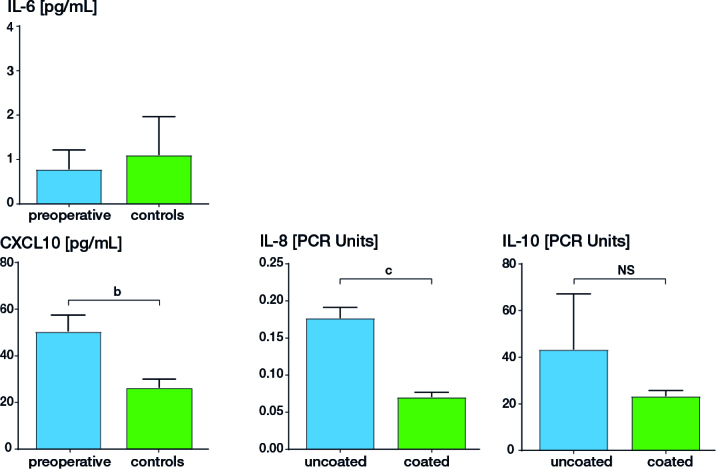
Relative expression levels of mRNA for IL-10 and IL-8 in peripheral blood of patients with the uncoated (n = 98) and coated (n = 92) TKR (e.motion/e.motion-AS). **^c^** p < 0.001; standard deviation is given. NS: not significant.

The statistically significant difference of the IL-8 content in the serum samples of arthroplasty patients was paralleled by a significant difference in the IL-8 expression on RNA level (uncoated: 0.18 [SD 0.02] vs. coated: 0.09 [SD 0.007]). RNA analysis was also done for Foxp3 and IL-10 with the intention to assess regulatory cell marker, for a caspase (casp1) responsible for cleaving pro IL-1I into its active form and for IFN©. Expression of all 4 of these was, however, similar in the 2 patient groups. The results of IL-8 and IL-10 expression are shown in Figure 2.

## Discussion

It is believed that after wound healing and tissue regeneration most arthroplasty patients adapt to the implant and regain quality of life. However, tissue homeostasis around the “foreign body” may be disturbed by inflammatory mediator response to wear and metal ions. Discussion was triggered by some patients developing extreme adverse reactions to metal-on-metal hip arthroplasty, i.e., pseudotumor formation (Hutt et al. 2016). JКmsen et al. (2017) described the correlations between macrophage polarizing cytokines, osteoclast activity, and toll-like receptors—particularly TLR4 in tissue around aseptically loosened hip implants. Among other things, they reported that IL-8 expression correlates with early revision time. Apart from potential toxic or unspecific immunostimulatory mechanisms, metal allergy could also play a role since enhanced metal sensitivity has been found in patients with failed arthroplasty (Hallab et al. 2001, Thomas et al. 2009, Granchi et al. 2012). Thus, use of alternative materials or surface-coated CoCrMo alloys could be an alternative for metal-sensitive patients (Bader et al. 2008). In our study we describe comparable good clinical 5-year results in patients with the same CoCrMo-based TKR in uncoated and surface-coated versions. The second major conclusion of our study is that patients with uncoated, well-performing TKR nevertheless present with a subclinical proinflammatory cytokine pattern in the peripheral blood. Unexpectedly, we found that serum IL-8 was substantially elevated in standard uncoated TKR patients. Since in center 1 the surface-coated variant of TKR was used in all patients, the patient series consisted of 6/97 with and 91/97 without known metal allergy. This approach avoids a selection bias regarding metal-allergic patients. The clinical performance reflected by the KSS score was almost identical. This is in line with the Kaplan–Meier implant survival rate of 98% (coated) and 97% (uncoated). These numbers are in accordance with published data on 5-year survival rates of TKR ranging between 88 and 98% (Lee et al. 2013).

The rationale for determination of selected cytokines and chemokines in peripheral blood was: first, to avoid joint aspirates in symptom-free patients and second, systemic cytokine pattern in peripheral blood is described to reflect a variety of local tissue conditions (for example rheumatoid arthritis, malignant tumor, hepatic or lung inflammatory disease) (Fang et al. 2016, Lin et al. 2016, Matz et al. 2016, Tan et al. 2017).

We selected an array of partly innate proinflammatory mediators (IL-1I, IL-4, IL-5, IL-6, IL-8), the specific T-cell chemoattractant CXCL10 and the counterregulatory immune “dampening” IL-10. Also, when comparing with the “normal” situation, i.e., osteoarthritis patients prior to knee arthroplasty and healthy individuals without osteoarthritis and without implant, a significantly higher blood level of IL-8 was found in patients with uncoated CoCrMo-based TKR. Despite an allergy background being overrepresented in the 20 preoperative controls, this did not influence the “unspecific blood cytokine profile”. In addition, molecular analysis of peripheral blood cells showed enhanced expression of IL-8 in the patients with uncoated TKR. When considering conditions with potential immune activation/production of systemic proinflammatory mediators, elevated IL-8 levels are typically found in various settings: exercise stimulates increases in the circulating concentrations of several cytokines, namely IL-8 (Peake et al. 2015); in peripheral arterial disease acute phase proteins, matrix metalloproteases, and cytokines—in particular IL-8—are considered as potential biomarkers of pathogen-related inflammation (Signorelli et al. 2014); the degree of elevated serum IL-8 is related to the extent of potential inflammation and fibrosis in chronic liver disease (Nobili et al. 2004); elevated serum IL-8 levels are found for several types of cancer and are discussed as a prognostic marker for malignant disease (Palena et al. 2012, Jin et al. 2014). Already in 2005 Tanaka et al. had reported two observations: serum and synovial fluid IL-8 levels were significantly correlated in hip arthroplasty patients—and elevated IL-8 levels correlated with aseptic loosening of hip arthroplasty (Tanaka et al. 2005). 12 years later, in 2017 JКmsen et al. in retrieval tissue samples not only observed the “protective” IL-4 polarized M2 macrophage type and the IFN© governed M1 macrophage type adding to aseptic loosening, but also found that “IL-8 expression correlates with early revision time” (JКmsen et al. 2017).

Metal ions, in particular cobalt, greatly enhance in vitro IL-8 production by various cell types (Ninomiya et al. 2013, Lawrence et al. 2014). Recent investigations have shown that cobalt and nickel induce such IL-8 expression via TLR4 activation (Anjum et al. 2016, Lin et al. 2016). In addition, as reported by Lawrence et al. (2014), cobalt ions promote the expression of CXCL10, a T-lymphocyte chemokine. Similarly to IL-6 (Wang et al. 2014, Ettinger et al. 2015) as contributor to autoimmune and inflammatory diseases, the chemokine IL-8 has multiple proinflammatory effects. As an intravascular danger signal it enables trafficking of leukocytes to sites of tissue injury (by chemotaxis and expression of vascular adhesion molecules), it promotes neovascularization, it enables epithelial–mesenchymal transition (both in wound healing/tissue regeneration and fibrosis/cancer progression) and it prolongs ongoing inflammation. With regard to prolonged peri-implant inflammatory response, Anjum et al. (2016) presented an interesting working model. Cobalt ions released from arthroplasty activate TLR4 on immune cells and endothelial cells, resulting in cytokine and chemokine (including IL-8) release that leads among others to inflammatory cell recruitment from the circulation.

Were our patients with “standard”, i.e., uncoated, TKR and high IL-8 levels at risk of earlier revision? When planning the study—yet unaware of such results—we had wondered if also some specific, i.e., T-cell related, marker or cytokines reflecting regulatory mechanism were demonstrable in our patients. For the latter aspects we assessed IL-10 for the following reasons: Martire-Greco et al. (2013) had reported that addition of IL-10 reduced LPS-induced TNF↑- and IL-8 secretion of human neutrophils in vitro; IL-10 was found to reflect a potent anti-inflammatory, immune-dampening response for example in pregnancy allowing “fetal tolerance” (Santner-Nanan et al. 2013), in successful immunotherapy with pollen allergens (Jutel and Akdis 2011) or symptom-free metal (titanium) dental implant patients (Thomas et al. 2013). Apart from its tolerogenic role IL-10 can also play a role in immune-mediated diseases by promoting autoantibody production and cytotoxic T-cell responses (Geginat et al. 2016). Even if we tend to consider the here observed IL-10 production as immunoregulatory, such a pivotal role of IL-10 together with enhanced IL-8 production needs attention in future investigations. Cassuto et al. (2017) have recently reported their results of repeated measuring of plasma biomarkers in hip arthroplasty over a period of almost 2 decades. Among their findings, there was a striking peak of IL-8 around 5–6 years post-surgery and a late rise of RANKL. In this study the focus was set on bone remodeling/healing phenomena. Nevertheless such a late proinflammatory mediator peak accords with our findings. Unfortunately IL-10 levels were not assessed by Cassuto et al. (2017).

Since CXCL10 is described as a chemoattractant for the recruitment of activated T-cells (Wang et al. 2016), i.e., thus prolonging or increasing adaptive immune response, we assessed its serum levels as well. The 2 arthroplasty patient groups had more elevated levels with the highest, but statistically non-significant, increase in uncoated arthroplasty. Expression of Foxp3 as a marker of regulatory T-cells could again be found in both patient groups, with not significantly higher values in the uncoated TKR group.

Immune reactivity to implanted metal arthroplasty is an area of growing research intensity. At the same time the understanding of immunological responses to it and of cytokine homeostasis in such patients still remains incomplete. Our study, to our knowledge, is the first comparative analysis of serum cytokine responses and clinical performance in patients with standard and surface-coated TKR. The observed differences, i.e., the proinflammatory yet clinically inapparent and controlled condition in the standard TKR patient, should trigger further work to identify additional factors contributing to implant failure in given patients. Subsequent studies should include blood sampling at more time points and also follow-up study examinations with the 10- and 15-year results should be planned.

In summary, we could observe differences in cytokine patterns between patients with coated and uncoated knee joint implants but with similar clinical outcomes at the 5-year follow-up. Identifying the complex interplay of individual “bio-mediators” may add to the understanding of why implants might fail. Thus, the long-term cumulative effect of those cytokine changes should be a matter for further research.

The authors thank Prof. Lindenmayr for initiating the coated TKA concept at Memmingen Hospital.

TP: study leader, manuscript preparation; PH, HK, US, AO, DA: clinical work, patient recruitment, and examination (in the different study centers); SB: cytokine measurement, data analysis, statistics; SC: clinical work, patient examination, manuscript discussion.

*Acta* thanks Christian P Delaunay and Stephan SЪder for help with peer review of this study.

**Table:** Characteristics of the 196 patients with primary TKR and of the 40 individuals without metal implant

	Group I uncoated**^a^**	Group II coated**^b^**	Group III preoperative**^c^**	Group IV healthy controls**^d^**
Patients	99	97	20	20
Sex (female/male)	54/45	56/41	17/3	14/6
Age median (years)	67 (44–84)	70 (48–82)	60 (46–84)	61 (52–63)
Smoker (formely smokers)	5 (11)	10 (19)	2	2
History of skin metal allergy	0	6	14	3
Atopy^e^	11	4	12	3

**^a^** Uncoated = primary knee arthroplasty with e.motion.

^b^ Coated = primary knee arthroplasty with multilayer coated, i.e., AS e.motion.

^c^ Gonarthrosis patients, prior to knee arthroplasty (at present no metal implant)

^d^ Healthy patients without metal implant.

^e^ Atopy = history of hay fever and/or allergic asthma and/or atopic dermatitis.
